# APE1/Ref-1 Inhibits Phosphate-Induced Calcification and Osteoblastic Phenotype Changes in Vascular Smooth Muscle Cells

**DOI:** 10.3390/ijms18102053

**Published:** 2017-09-25

**Authors:** Ki Mo Lee, Eun Ok Lee, Yu Ran Lee, Hee Kyoung Joo, Myoung Soo Park, Cuk-Seong Kim, Sunga Choi, Jin-Ok Jeong, Byeong Hwa Jeon

**Affiliations:** 1 Research Institute of Medical Sciences, Department of Physiology, School of Medicine, Chungnam National University, 266 Munhwa-ro, Jung-gu, Daejeon 35015, Korea; lkm4703@naver.com (K.M.L.); y21c486@naver.com (E.O.L.); lyr0913@cnu.ac.kr (Y.R.L.); hkjoo79@cnu.ac.kr (H.K.J.); nova38@hanmail.net (M.S.P.); cskim@cnu.ac.kr (C.-S.K.); sachoi@cnu.ac.kr (S.C.); 2 Division of Cardiology, Department of Internal Medicine, Chungnam National University, Daejeon 35015, Korea; jojeong@cnu.ac.kr

**Keywords:** APE1/Ref-1, inorganic phosphate, vascular smooth muscle cells, vascular calcification

## Abstract

Vascular calcification plays a role in the pathogenesis of atherosclerosis, diabetes, and chronic kidney disease; however, the role of apurinic/apyrimidinic endonuclease 1/redox factor-1 (APE1/Ref-1) in inorganic phosphate (Pi)-induced vascular smooth muscle cell (VSMC) calcification remains unknown. In this study, we investigated the possible role of APE1/Ref-1 in Pi-induced VSMC calcification. We observed that Pi decreased endogenous APE1/Ref-1 expression and promoter activity in VSMCs, and that adenoviral overexpression of APE1/Ref-1 inhibited Pi-induced calcification in VSMCs and in an ex vivo organ culture of a rat aorta. However, a redox mutant of APE1/Ref-1(C65A/C93A) did not reduce Pi-induced calcification in VSMCs, suggesting APE1/Ref-1-mediated redox function against vascular calcification. Additionally, APE1/Ref-1 overexpression inhibited Pi-induced intracellular and mitochondrial reactive oxygen species production, and APE1/Ref-1 overexpression resulted in decreased Pi-induced lactate dehydrogenase activity, pro-apoptotic Bax levels, and increased anti-apoptotic Bcl-2 protein levels. Furthermore, APE1/Ref-1 inhibited Pi-induced osteoblastic differentiation associated with alkaline phosphatase activity and inhibited Pi-exposure-induced loss of the smooth muscle phenotype. Our findings provided valuable insights into the redox function of APE1/Ref-1 in preventing Pi-induced VSMC calcification by inhibiting oxidative stress and osteoblastic differentiation, resulting in prevention of altered osteoblastic phenotypes in VSMCs.

## 1. Introduction

Vascular calcification described abnormal calcium deposition in vessel walls and is a characteristic feature of atherosclerosis, diabetes mellitus, and end-stage renal disease. Vascular calcification is linked to plaque instability and contributes to increased cardiovascular mortality and poor cardiovascular outcomes for patients. Aortic calcification is an important marker in atherosclerosis, and chronic inflammatory diseases, such as atherosclerosis, also contribute to the progression of calcification [1,2].

Apurinic/apyrimidinic endonuclease 1/redox factor-1 (APE1/Ref-1) is a multifunctional protein involved in base-excision DNA repair and transcriptional regulation of gene expression, as well as playing a pleiotropic role in controlling cellular responses to oxidative stress [3,4]. In particular, APE1/Ref-1 reduces intracellular production of reactive oxygen species (ROS) [3,4,5], and overexpression of APE1/Ref-1 suppresses tumour necrosis factor-α-induced monocyte adhesion to endothelial cells [6] and inhibits hypoxia-induced endothelial-cell apoptosis [7]. Moreover, APE1/Ref-1 inhibits balloon-injury-induced neointimal formation in rats [8], suggesting that it exhibits anti-inflammatory functions in the vascular endothelium.

Vascular smooth muscle cells (VSMCs) can undergo a phenotype change during development [9]. The contractile phenotype of VSMCs is regulated by a balance of several transcriptional factors and can change to support repair processes or return to a normal phenotype. This phenotype change is referred to as phenotypic plasticity or VSMC transition; however, inappropriate phenotype changes occurring under certain conditions, such as high inorganic phosphate (Pi) concentrations, induce vascular calcification via modulation of osteogenic transcription factors [10].

Several transcription factors, including nuclear factor-kappa B (NF-κB) and activator protein-1 (AP-1), are activated by redox regulation of APE1/Ref-1 [11]. Redox signalling associated with APE1/Ref-1 is closely related to cell differentiation and senescence [12]. In particular, inhibition of APE1/Ref-1-mediated redox signalling increases the osteoblastic differentiation of dental papilla cells [13]. However, it remains unknown whether APE1/Ref-1 affects vascular calcification and in what context. Here, we tested the hypothesis that APE1/Ref-1 suppresses vascular calcification and investigated the role and mechanism of APE1/Ref-1 in Pi-induced calcification in VSMCs and rat aorta.

## 2. Results

### 2.1. Pi Inhibits APE1/Ref-1 Transcription and Protein Expression

**Figure 1:**
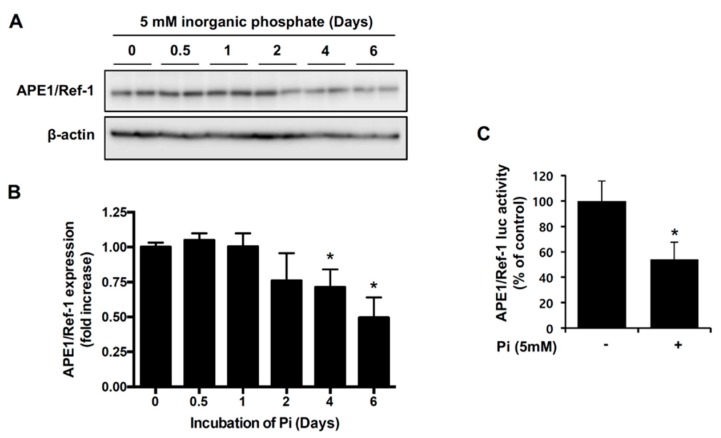
Inorganic phosphate (Pi) inhibited apurinic/apyrimidinic endonuclease 1/redox factor-1 (APE1/Ref-1) transcription and protein expression in vascular smooth muscle cells (VSMCs). (**A**,**B**) Changes in endogenous APE1/Ref-1 expression during cell exposure to Pi (5 mM) for the indicated times (0–6 days). Expression of APE1/Ref-1 and β-actin was measured by Western blot. Typical representative data (**A**) and summarized data (**B**) are shown. Each bar represents the mean ± standard error of the mean (SEM) fold increase relative to basal APE1/Ref-1 expression (*n* = 5). * *p* < 0.05 vs. control by one-way analysis of variance (ANOVA). (**C**) Effect of Pi (5 mM) on APE1/Ref-1-promoter luciferase activity in primary VSMCs. Firefly luciferase activities were normalized to those of the co-transfected *Renilla* luciferase. Cells were treated with Pi (5 mM) for 4 days. Each bar represents the mean ± SEM as a percentage of basal APE1/Ref-1-promoter luciferase activity (*n* = 6). * *p* < 0.05 vs. control by one-way ANOVA.

A Pi challenge with ascorbic acid can induce vascular smooth muscle calcification [14]. First, we investigated the effect of Pi on endogenous APE1/Ref-1 expression in VSMCs. As shown in Figure 1A, when cells were incubated with 5 mM Pi for 0.5 to 6 days, endogenous APE1/Ref-1 protein expression was unchanged upon Pi exposure for 2 days, but significantly reduced upon Pi exposure for 4 to 6 days. To determine whether Pi-mediated downregulation of APE1/Ref-1 occurred at the promoter level, cells were transfected with an APE1/Ref-1-promoter–luciferase reporter construct (−4000/+65 APE1/Ref-1–Luc, containing the APE1/Ref-1-promoter sequence comprising an area from −4000 bp to +65 bp of the gene) and incubated with 5 mM Pi for 4 days. As shown in Figure 1B, APE1/Ref-1-promoter activity was reduced to ~54% upon Pi exposure for 4 days, suggesting inhibition of APE1/Ref-1 transcription.

### 2.2. APE1/Ref-1 Suppresses Pi-Induced Calcification in VSMCs

**Figure 2:**
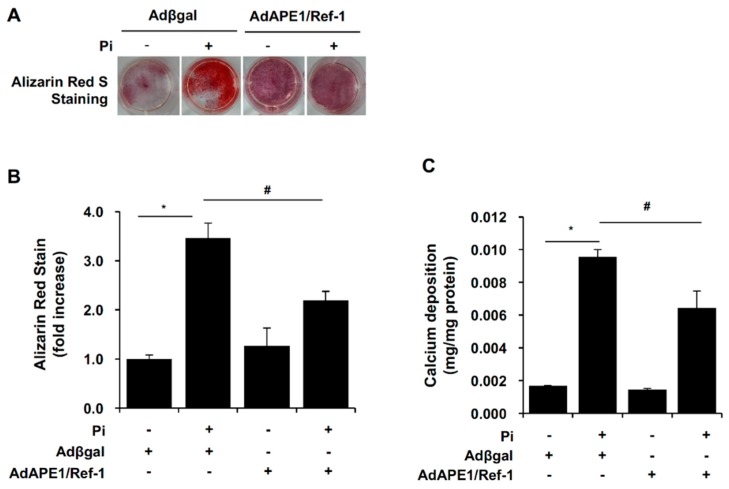

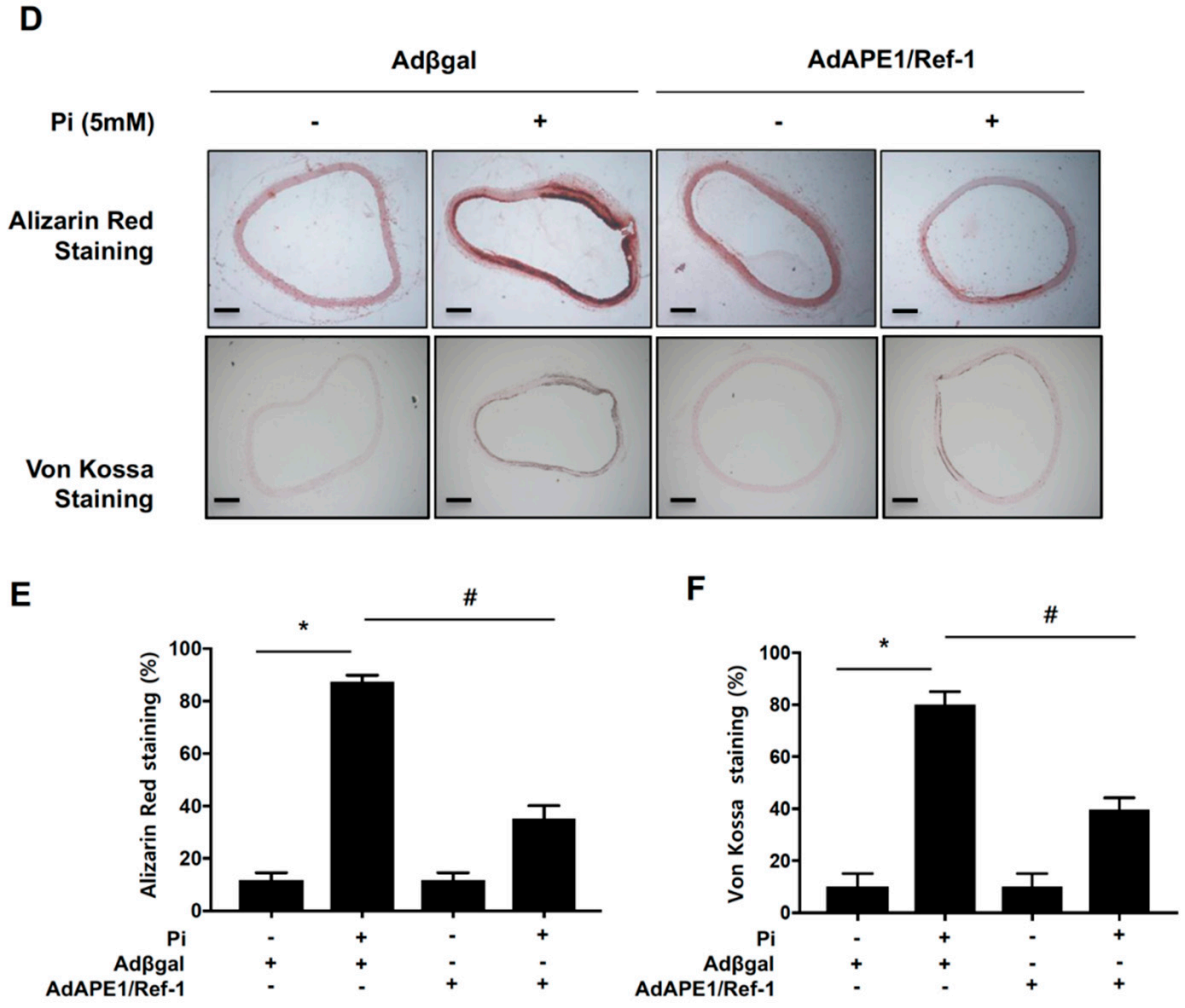
APE1/Ref-1 overexpression inhibited inorganic phosphate (Pi)-induced vascular calcification in vascular smooth muscle cells (VSMCs) (**A**–**C**) and ex vivo aorta (**D**–**F**). (**A**) Representative Western blot for APE1/Ref-1. After adenoviral APE1/Ref-1 infection at 90% confluency for 24 h, VSMCs were treated with Pi (5 mM) for 6 days. (**B**) Representative images (top) and quantification of alizarin red staining. At the end of incubation, cells were stained with 2% alizarin red S. Each bar represents the mean ± standard error of the mean (SEM) (*n* = 3). * *p* < 0.05 vs. control; ^#^ *p* < 0.05 vs. Pi alone by one-way ANOVA. (**C**) Calcium-deposition assay using the same conditions as described in (**B**). To evaluate calcium deposition, cells were decalcified by incubation for 24 h with 0.6 N HCl. Calcium content in the supernatant was determined colourimetrically using *O*-cresolphthalein and normalized to the protein concentration as described in the Materials and Methods. Each bar represents the mean ± SEM (*n* = 5). * *p* < 0.05 vs. control; ^#^ *p* < 0.05 vs. Pi alone by one-way ANOVA. (**D**–**F**) APE1/Ref-1 overexpression inhibited Pi (5 mM)-induced aortic vascular calcification ex vivo. After adenoviral APE1/Ref-1 (10^9^ plaque-forming units in 2% Dulbecco’s modified Eagle medium) transfection, aortic tissues without the endothelium were treated with Pi (5 mM) for 6 days. Calcification of aortic tissue was assayed by alizarin red staining and von Kossa staining. (**D**) Representative data of aortic calcification. Quantitative analysis of alizarin red staining (**E**) and von Kossa staining (**F**). Each bar represents the mean ± SEM (*n* = 4). * *p* < 0.05 vs. control; ^#^ *p* < 0.05 vs. Pi alone by one-way ANOVA. Microscopes at 40× magnification were used for visualization. Scale bar: 100 μm.

To determine the role of APE1/Ref-1 in Pi-induced VSMC calcification, we studied the effect of adenoviral overexpression of APE1/Ref-1 (200 multiplicity of infection (MOI)). Adenovirus-transfected cells were exposed to Pi (5 mM) in the presence of 10% fatal bovine serum (FBS) for 6 days, and APE1/Ref-1 overexpression in VSMCs was confirmed by Western blot analysis (Figure 2A). To evaluate Pi-induced VSMC calcification, we performed alizarin red staining and a calcium-deposition assay. Under basal normal phosphate conditions, vascular calcification was not induced according to the absence of alizarin red staining and the level of calcium deposition (Figure 2B,C). However, as expected, vascular calcification was induced under high-Pi conditions (5 mM Pi for 6 days). Adenoviral APE1/Ref-1 overexpression (200 MOI) did not induce vascular calcification; however, APE1/Ref-1 overexpression markedly inhibited Pi-induced VSMC calcification assessed by alizarin red staining and calcium-deposition assay (Figure 2B,C). Recently, it was reported that vascular calcification was induced by Pi exposure in an organ culture of the aorta [15]. To investigate whether APE1/Ref-1 inhibits Pi-induced VSMC calcification in an organ culture of the aorta, thoracic aortas were removed from rats (*n* = 4) and cultured in a medium containing Pi (5 mM) for 6 days. Aortic ring segments were incubated with APE1/Ref-1 (1 × 10^9^ plaque-forming units (pfu)) in Dulbecco’s modified Eagle medium (DMEM) containing 2% FBS for 24 h, and vascular calcification was assessed by alizarin red/von Kossa staining. Under basal phosphate conditions, no calcification was observed in organ-cultured aortas; however, calcification was observed in endothelial and vascular smooth muscle layers of the aorta upon Pi exposure (Figure 2D). Interestingly, adenoviral overexpression of APE1/Ref-1 significantly suppressed Pi-induced aortic calcification (alizarin red staining) and phosphate precipitation (von Kossa staining) in the ex vivo system (Figure 2E,F).

### 2.3. APE1/Ref-1 Gene Silencing Aggravates Pi-Induced Calcification

**Figure 3:**
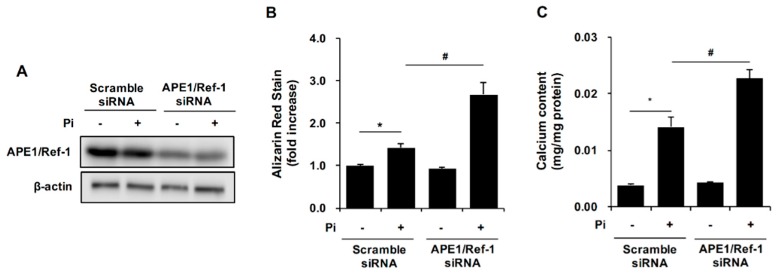
Gene silencing of APE1/Ref-1 increased inorganic phosphate (Pi)-induced calcification in vascular smooth muscle cells (VSMCs). (**A**) Representative Western blot results of APE1/Ref-1 gene silencing. After transfection with small-interfering (si)RNA for APE1/Ref-1, VSMCs were incubated with Pi (5 mM) for 4 days; (**B**) At the end of incubation, cells were stained with 2% alizarin red S. Each bar represents the mean ± standard error of the mean (SEM) (*n* = 5). * *p* < 0.05 vs. control; ^#^ *p* < 0.05 vs. Pi alone by one-way ANOVA; (**C**) Calcium-deposition assay using the same conditions as described in (**B**). To evaluate calcium deposition, cells were decalcified by incubation for 24 h with 0.6 N HCl. The calcium content in the supernatant was determined colourimetrically using *O*-cresolphthalein and normalized to the protein concentration as described in the Materials and Methods. Each bar represents the mean ± SEM (*n* = 5). * *p* < 0.05 vs. control; ^#^ *p* < 0.05 vs. Pi alone by one-way ANOVA.

**Figure 4:**
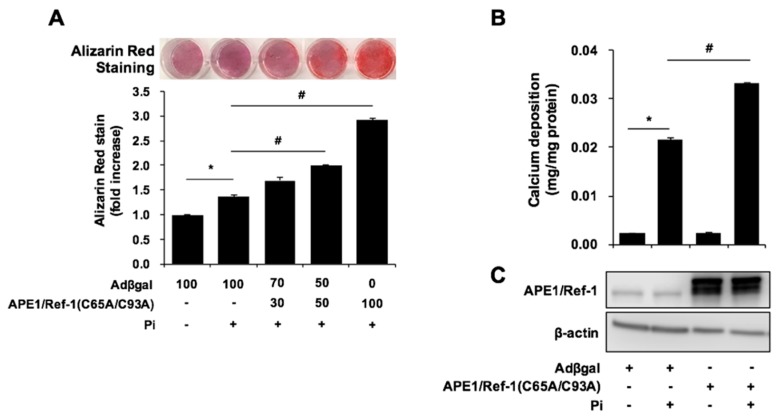
The APE1/Ref-1 redox mutant does not suppress inorganic phosphate (Pi)-induced calcification in vascular smooth muscle cells. (**A**) Alizarin red staining. (**B**) Calcium-deposition assay. Cells were transfected with APE1/Ref-1 C65A/C93A at a multiplicity of infection from 30 to 100 and exposed to Pi (5 mM) for 6 days. Each bar represents the mean ± standard error of the mean (SEM) (*n* = 6). * *p* < 0.05 vs. control; ^#^ *p* < 0.05 vs. Pi alone by one-way ANOVA. (**C**) After adenoviral transfection, overexpression of APE1/Ref-1 C65A/C93A was confirmed by Western blot analysis. The redox mutant of hemagglutinin-tagged APE1/Ref-1 C65A/C93A was ~40 kDa, which was larger than that of endogenous APE1/Ref-1 (37 kDa).

To confirm the function of endogenous APE1/Ref-1 in Pi-induced VSMC calcification, cells were transfected with small-interfering (si)RNA for APE1/Ref-1, followed by analysis of the effect of APE1/Ref-1 silencing on Pi-induced VSMC calcification. APE1/Ref-1 siRNA-transfected cells were exposed to Pi (5 mM) in the presence of 10% FBS for 4 days, and silencing of APE1/Ref-1 expression in VSMCs was confirmed by Western blot analysis (Figure 3A). APE1/Ref-1 protein expression decreased by ~50% following transfection of APE1/Ref-1 siRNA for 4 days. As shown in Figure 4B,C, APE1/Ref-1 downregulation increased Pi-induced VSMC calcification and calcium deposition, suggesting a preventive role for endogenous APE1/Ref-1 against vascular calcification.

### 2.4. APE1/Ref-1 Redox Mutants Are Unable to Suppress Pi-Induced VSMC Calcification

The redox-regulatory domain of APE1/Ref-1 is characterized by two critical cysteine residues (C65 and C93) that are critical for APE1/Ref-1-specific anti-inflammatory activity in endothelial cells [16,17]. To determine the role of APE1/Ref-1 redox functions, we investigated the effect of adenoviral overexpression of APE1/Ref-1 redox mutants (C65A/C93A) generated by substitutions of C65 and C93 with alanine. Adenoviral overexpression of APE1/Ref-1 C65A/C93A did not suppress Pi-induced calcification, but the loss of the reducing residues instead accelerated this process, as shown by alizarin red staining (Figure 4A) and by calcium-deposition assay (Figure 4B). This result indicated that the redox mutant of APE1/Ref-1 was unable to suppress Pi-induced VSMC calcification, and that C65 and C93 in the redox active site of APE1/Ref-1 contributes to the prevention of Pi-induced VSMC calcification.

### 2.5. APE1/Ref-1 Suppresses Pi-Induced ROS Production and Apoptosis

**Figure 5:**
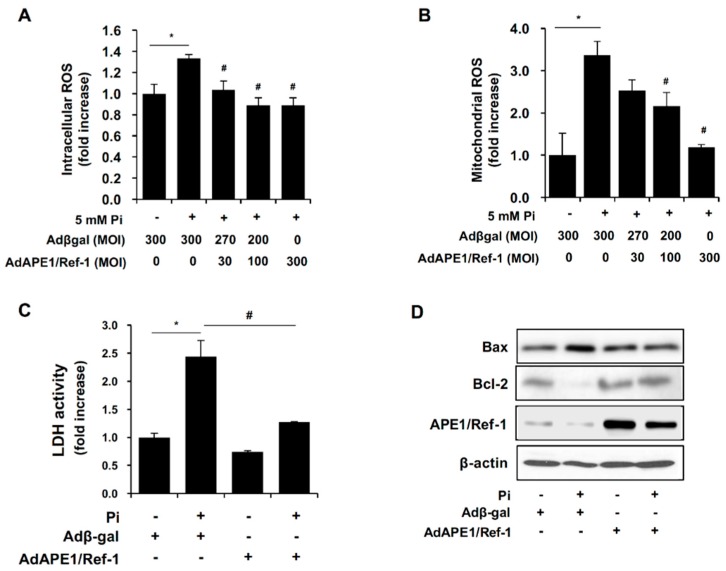
APE1/Ref-1 overexpression inhibits inorganic phosphate (Pi)-induced reactive oxygen species (ROS) production and apoptosis in vascular smooth muscle cells (VSMCs). (**A**) The level of intracellular ROS was measured using 2′,7′-dichlorofluorescin diacetate, and (**B**) the level of mitochondrial ROS was determined using MitoSOX, mitochondrial superoxide indicator. Cells were treated with Pi (5 mM) for 1 day, and VSMCs were infected with an adenovirus encoding APE/Ref-1 at a multiplicity of infection (MOI) from 30 to 300. Adβgal at the same MOI was used as a control. Each bar represents the mean ± standard error of the mean (SEM) fold increase relative to basal ROS production (*n* = 6). * *p* < 0.05 vs. control; ^#^ *p* < 0.05 vs. Pi alone by one-way ANOVA. (**C**) Lactate dehydrogenase (LDH) activity in the cell-culture medium. (**D**) APE1/Ref-1 inhibited Pi-induced apoptosis in VSMCs. Changes in Bax and Bcl-2 expression in VSMCs were evaluated by Western blot analysis. VSMCs were infected with an adenovirus encoding APE1/Ref-1 at an MOI of 300, with Adβgal at the same MOI used as a control. Similar results were observed in replicate experiments, and representative data is shown.

Because oxidative stress is related to the pathogenesis of VSMC calcification, we investigated whether APE1/Ref-1 suppresses Pi-induced ROS production in VSMCs. Adenoviral APE1/Ref-1 at 30 MOI to 300 MOI was transfected into VSMCs, which were then incubated with Pi (5 mM) for 1 day. Pi exposure significantly increased intracellular ROS (assessed with 2′,7′-dichlorofluorescin diacetate (H_2_DCFDA); Figure 5A) and mitochondrial ROS (assessed with MitoSOX; Figure 5B) levels; however, Pi-induced intracellular and mitochondrial ROS production were inhibited by APE1/Ref-1 overexpression in an MOI-dependent manner. To investigate whether APE1/Ref-1 inhibits cell death and the expression of genes promoting apoptosis, cytotoxicity was determined by measuring lactate dehydrogenase (LDH) activity in cell-culture medium, and Western blot analysis was used to determine the expression of Bax and Bcl-2 proteins. As shown in Figure 5C, Pi-exposure increased LDH activity, which was inhibited by APE1/Ref-1 overexpression. Furthermore, APE1/Ref-1 inhibited Bax expression and prevented the loss of Bcl-2 expression in response to Pi exposure, suggesting that APE1/Ref-1 exhibits anti-oxidant and/or anti-apoptotic activity against Pi-induced calcification. 

### 2.6. APE1/Ref-1 Suppresses Pi-Induced Alkaline Phosphatase (ALP) Activity and Osteoblastic Differentiation

**Figure 6:**
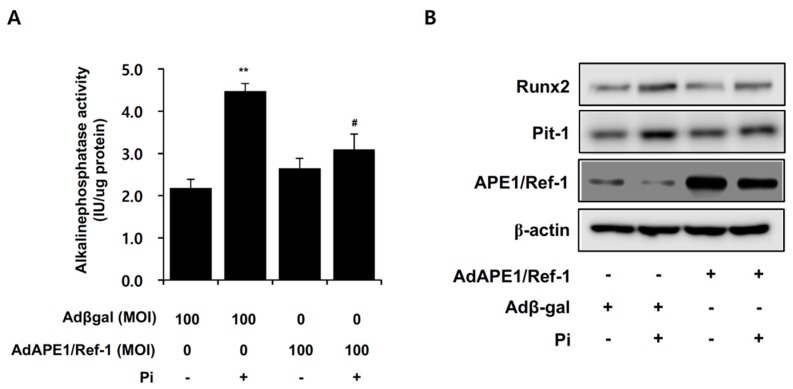
APE1/Ref-1 overexpression inhibits inorganic phosphate (Pi)-induced alkaline phosphatase (ALP) activity and Runx-2 and Pit-1 expression in vascular smooth muscle cells (VSMCs). (**A**) ALP activity was determined. At the end of incubation, cells were washed with phosphate-buffered saline and solubilized with 1% Triton X-100 in 0.9% NaCl, and ALP activity was normalized to protein concentration. Cells were treated with Pi (5 mM) for 6 days, and VSMCs were infected with an adenovirus encoding APE1/Ref-1 at a multiplicity of infection (MOI) of 100, with Adβgal used at the same MOI as a control. Each bar represents the mean ± standard error of the mean (*n* = 5). ** *p* < 0.01 vs. control; ^#^ *p* < 0.05 vs. Pi alone by one-way ANOVA; (**B**) APE1/Ref-1 inhibited Pi-induced Runx2 and Pit-1 expression in VSMCs. Changes in Runx2 and Pit-1 expression in VSMCs were evaluated by Western blot analysis. VSMCs were infected with an adenovirus encoding APE/Ref-1 at an MOI of 300, with Adβgal used at the same MOI as a control. Similar results were observed in replicate experiments, and representative data is shown.

ALP promotes calcification by providing Pi to promote mineralization and is a potential therapeutic target against VSMC calcification [18]. To determine whether APE1/Ref-1 inhibits Pi-induced ALP activity, this activity was determined following overexpression of APE1/Ref-1, which significantly inhibited Pi-induced ALP activity (Figure 6A). Oxidative stress induces vascular calcification through modulation of osteogenic transcription factors, such as runt-related transcription factor 2 (Runx2) [10], and pituitary-specific positive transcription factor 1 (Pit-1) was identified as a major phosphate transporter in VSMCs and required for osteochondrogenic differentiation of smooth muscle cells [19]. Our results showed that Runx2 and Pit-1 expression increased following Pi exposure for 6 days; however, APE1/Ref-1 overexpression markedly inhibited Pi-induced Runx2 and Pit-1 expression, suggesting anti-osteoblastic activity from APE1/Ref-1.

### 2.7. AP1/Ref-1 Suppresses Pi-Induced Loss of VSMC Phenotype

**Figure 7:**
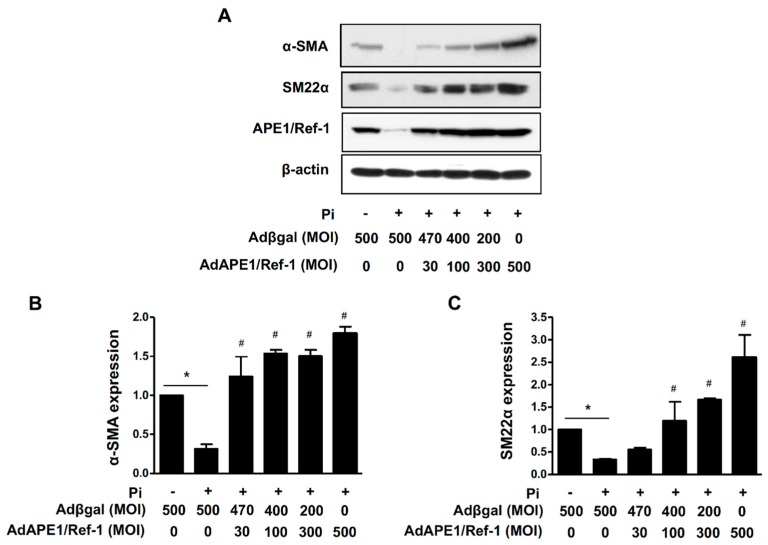
APE1/Ref-1 suppresses inorganic phosphate (Pi)-induced loss of the vascular smooth muscle cell phenotype. (**A**) Representative Western blot. (**B**,**C**) Quantified levels of α-SMA (**B**) and SM22α (**C**). Cells were transfected with APE1/Ref-1 at multiplicities of infection from 30 to 500 and exposed to Pi (5 mM) for 6 days. α-SMA and SM22α protein levels were analysed by Western blot. Each bar represents the mean ± standard error of the mean (*n* = 5). * *p* < 0.05 vs. control; ^#^ *p* < 0.05 vs. Pi alone by one-way ANOVA.

The contractile phenotype of the smooth muscle is defined as the expression of smooth muscle cell proteins, such as α-smooth muscle actin (α-SMA) and smooth muscle protein 22-α (SM22α), which is a calponin-related protein specifically expressed in adult smooth muscles [20]. We investigated the role of APE1/Ref-1 in the expression of VSMC-differentiation markers, such as α-SMA and SM22α. As shown in Figure 7, α-SMA and SM22α protein levels were significantly decreased by Pi (5 mM) exposure for 6 days; however, this loss of phenotype was recovered by APE1/Ref-1 overexpression. 

## 3. Discussion

In this study, we demonstrated a novel role for APE1/Ref-1 in Pi-induced vascular calcification and changes in VSMC phenotype and showed that the redox function of APE1/Ref-1 promoted inhibition of Pi-induced VSMC oxidative stress and vascular calcification. 

APE1/Ref-1 is ubiquitously expressed in cells, and its expression can be regulated at the transcriptional level [21]. Although APE1/Ref-1 gene expression is regulated by transcriptional modulation, several factors, including hormones or cytokines, can modulate APE1/Ref-1 expression. Furthermore, APE1/Ref-1 levels are closely related to adaptive or protective responses against the cytotoxic activity of oxidizing drugs [22]. A previous study reported reduced APE1/Ref-1 expression in neurons following transient global cerebral ischemia in rats [23]. Overexpression of the p53 tumour suppressor decreases APE1/Ref-1 expression in response to DNA damage and results in cellular apoptosis via binding of p53 to the APE1/Ref-1 promoter [24,25]. Additionally, hypoxia decreases APE1/Ref-1 levels, followed by significant apoptosis of human umbilical vein endothelial cells [7]. In the present study, chronic exposure to Pi for 6 days decreased APE1/Ref-1 protein expression and APE1/Ref-1-promoter-dependent activity. Therefore, these findings suggest that Pi-induced decreases in APE1/Ref-1 expression are strongly correlated with endothelial oxidative stress and apoptosis, resulting in vascular calcification in VSMCs. 

The basic nuclear functions of APE1/Ref-1 include regulation of transcription-factor activity, as well as DNA-repair functions. As an extranuclear function, APE1/Ref-1 inhibits intracellular ROS by inhibiting NADPH oxidase activation by Rac1 GTPase and vascular cell-adhesion molecule-1 expression in endothelial cells [3,16]. Additionally, APE1/Ref-1 exerts anti-inflammatory activities by reducing the activity of thiol–disulphide exchanges [26]. Gene silencing of APE1/Ref-1 expression decreases basal DNA-repair functions and redox activity, followed by increases in intracellular ROS production [27]. High levels of phosphate-induced apoptosis are associated with mitochondrial dysfunction, such as mitochondrial ROS production [28], with mitochondrial ROS promoting NF-κB p65 nuclear translocation, followed by phosphate-induced VSMC calcification [29]. In the present study, APE1/Ref-1 overexpression significantly inhibited Pi-induced ROS production and apoptosis, as well as vascular calcification of VSMCs in vitro and in rat aortas ex vivo. A recent study reported that vascular aldosterone synthase, which can be transcriptionally repressed by APE1/Ref-1, contributes to stimulation of osteochondrogenic transformation in human aortic vascular smooth muscle cells [30]. In the current study, gene silencing of APE1/Ref-1 increased Pi-induced vascular calcification in VSMCs; however, a double cysteine APE1/Ref-1 mutant (C65A/C93A) did not inhibit Pi-induced vascular calcification. These findings suggested that basal levels of endogenous APE1/Ref-1 exhibit inhibitory activity against vascular calcification, and that C65 and C93 of APE1/Ref-1 are important redox active sites against vascular calcification.

VSMCs show remarkable phenotype plasticity in response to environmental changes [31]. Phosphate induces calcification in VSMCs and causes phenotypic changes in VSMCs from a contractile to osteochondrogenic phenotype [32]. High levels of phosphates also induce VSMC apoptosis, which is a key regulator of VSMC calcification [33]. In human aortic smooth muscle cells, statins inhibit vascular calcification via the inhibition of apoptosis [34,35]. Furthermore, high levels of phosphate exposure induce changes in smooth muscle phenotype and result in vascular calcification of VSMCs.

Osteogenic differentiation of VSMCs is characterized by the expression of ALP and Runx2, and oxidative stress induces vascular calcification through modulation of osteogenic transcription factors, such as Runx2. Our data showed that APE1/Ref-1 overexpression markedly inhibited Pi-induced expression of Runx2 and the Pi transporter Pit-1 in VSMCs, suggesting that an anti-osteoblastic function for APE1/Ref-1 might be related to reduced oxidative stress. To investigate whether APE1/Ref-1 inhibits the loss of the smooth muscle phenotype, we examined the effects of APE1/Ref-1 overexpression on the smooth muscle phenotype markers α-SMA and SM22α. High levels of phosphates induced an almost complete loss of the smooth muscle phenotype, whereas APE1/Ref-1 overexpression strongly prevented the loss of the smooth muscle phenotype, suggesting a new target for the prevention of vascular calcification. High phosphate exposure is associated with increased DNA methyltransferase activity and methylation of the SM22α-promoter region [36], resulting in decreased SM22α protein expression and a loss of the smooth muscle cell phenotype. It remains unknown how APE1/Ref-1 inhibits loss of the smooth muscle phenotype in Pi-exposed VSMCs, although the reducing activity of APE1/Ref-1 in the presence of oxidative stress might be involved in preventing loss of the smooth muscle phenotype.

In conclusion, our study demonstrated a novel role for APE1/Ref-1 in Pi-induced vascular calcification and phenotype changes in VSMCs. APE1/Ref-1 inhibited Pi-induced vascular calcification, and the redox function of APE1/Ref-1 played an inhibitory role against Pi-induced vascular calcification by inhibiting osteoblastic differentiation and reducing oxidative stress. Uncovering the pathogenesis associated with vascular calcification will be helpful in the design of new target molecules or treatments targeting vascular calcification in chronic kidney diseases.

## 4. Materials and Methods

### 4.1. Chemicals and Reagents

Sodium phosphate (NaH_2_PO4 and Na_2_HPO4), ascorbic acid, alizarin red S, cetylpyridinium chloride, silver nitrate, ethylenediaminetetraacetic acid, ethylene glycol-*bis*(β-aminoethyl ether)-*N*,*N*,*N*′,*N*′-tetraacetic acid, NP-40, and sodium deoxycholate were purchased from Sigma-Aldrich (St. Louis, MO, USA). Penicillin-streptomycin antibiotic mixture and FBS were purchased from Life Technologies (Grand Island, NY, USA). DMEM and Hanks’ balanced salt solution (HBSS) were purchased from Welgene (Daegu, Korea). Phosphatase- and protease-inhibitor cocktails were purchased from Roche Diagnostics (Manheim, Germany). Antibodies specific for Bax, Bcl-2, and α-SMA were purchased from Cell Signaling Technology (Beverly, MA, USA). Antibodies against Runx2 and Pit-1 were obtained from Proteintech Group (Chicago, IL, USA). Anti-β-actin and anti-SM22α were obtained from Sigma-Aldrich and Abcam (Cambridge, MA, USA), respectively. A polyclonal APE1/Ref-1 antibody generated by immunizing rabbits with recombinant human APE1/Ref-1 was prepared in our laboratory [26].

### 4.2. Primary VSMC Isolation and Cell Culture

VSMCs were obtained from the thoracic aortas of 8-week-old male Sprague–Dawley rats using a tissue explant and enzymatic digestion method [37]. Briefly, rats were euthanized with ketamine (80 mg/kg; intraperitoneal (i.p.)) and xylazine (12 mg/kg; i.p.), the aorta was dissected, and the adventitia was carefully removed with forceps. The aorta was then cut open longitudinally, and the endothelium was gently scraped off with a scalpel. The aorta was cut into squares of ~1 mm^2^, which were incubated with 0.25% trypsin at 37 °C for 10 min to remove any remaining adventitia and endothelium. Aorta fragments were placed lumen side down in a tissue-culture flask and cultured in DMEM supplemented with 20% FBS at 37 °C. VSMCs that migrated from the explants were collected and maintained in DMEM containing 10% (*v*/*v*) FBS and 1% (*v*/*v*) penicillin–streptomycin at 37 °C in a humidified atmosphere containing 5% CO_2_. Cells used in experiments were from passages six to eight. The purity of VSMCs was confirmed by immunocytochemical staining based on positive staining with anti-α-SMA. Animal experiments were approved by the Animal Ethic Committee of Chungnam National University (CNU-00221) and conformed to NIH guidelines for the care and use of laboratory animals.

### 4.3. Induction of VSMC Calcification In Vitro

VSMC calcification was induced according to methods reported previously [14]. Briefly, when confluent cells were incubated in a calcification medium consisting of DMEM supplemented with 10% FBS, 5 mM Pi (a mixture of NaH_2_PO_4_ and Na_2_HPO_4_ (pH 7.4)), and 50 μg/mL ascorbic acid. The medium was replaced with fresh medium every 2 to 3 days for 6 days. 

### 4.4. Adenoviral Transfections

Adenoviruses encoding full-length APE1/Ref-1 (AdAPE1/Ref-1) and a double redox point mutant (C65A/C93A) were used as described previously [16,38]. Cells were infected at the indicated MOI for 24 h. The total adenoviral transfection was balanced using the same MOI of Adβgal as a control adenovirus. 

### 4.5. APE1/Ref-1 siRNA

Rat siRNA and scrambled siRNA were purchased from Bioneer Co. (Daejeon, Korea). VSMCs were transfected with 20 nM chemically synthesized siRNA targeting rat APE1/Ref-1 (5′-GUC UGG UAA GAC UGG AGU ACC-3′) and scrambled siRNA, which was used as a control, using Lipofectamine RNAiMAX according to manufacturer instructions (Invitrogen, San Diego, CA, USA). One day after the transfection, VSMCs were challenged with the calcification medium, which was replaced with fresh medium every 2 days for 4 days, followed by their use for subsequent experiments.

### 4.6. Evaluation of VSMC Calcification

Alizarin red S staining was used to assess the calcium deposition in VSMCs. Cells were fixed with 70% ethanol at 4 °C for 1 h, followed by staining with 2% alizarin red S (pH 4.2, adjusted with 1.0% NH_4_OH) for 10 min at room temperature. After staining, images were acquired using a Leica DM4000B microscope and DFC420 camera (Leica Microsystems SAS, Wetzlar, Germany). Stained cells were destained with 10% (*w*/*v*) cetylpyridinium chloride in 10 mM sodium phosphate (pH 7.0) for 15 min at room temperature. Absorbance of the released alizarin red was measured at 560 nm using an ELISA reader (Bio-Rad, Munich, Germany) and normalized to the protein concentration. To evaluate the calcium deposition, cells were decalcified by incubation for 24 h with 0.6 N HCl. The calcium content in the supernatant was determined colourimetrically using the *O*-cresolphthalein method and a calcium assay kit (Biovision, Mountain View, CA, USA). After decalcification, cells were washed three times with phosphate-buffered saline (PBS) and solubilized in a solution containing 0.1 N NaOH and 0.1% sodium dodecyl sulphate (SDS). The protein concentration was measured by the Bradford assay (Bio-Rad, Munich, Germany), and calcium content was normalized to the protein concentration. In some ex vivo experiments, von Kossa staining was used to assess the accumulation of Pi, which normally co-precipitates with calcium ions [39]. Aortic sections were treated with a 3% silver nitrate solution, and stained aortas were photographed under light microscopy. ALP activity was determined using the QuantiChrom alkaline phosphatase assay kit (Bioassay Systems, Hayward, CA, USA) according to manufacturer instructions. At the end of incubation, cells were washed with PBS and solubilized with 1% Triton X-100 in 0.9% NaCl. ALP activity was normalized to protein concentrations determined by the Bradford assay (Bio-Rad, Munich, Germany).

### 4.7. Western Blot Analysis

Protein lysates were obtained from cultured VSMCs using RIPA buffer containing 20 mM Tris–HCl (pH 7.5), 150 mM NaCl, 1 mM ethylenediaminetetraacetic acid, 1 mM ethylene glycol-*bis*(β-aminoethyl ether)-*N*,*N*,*N*′,*N*′-tetraacetic acid, 1% NP-40, 1% sodium deoxycholate, and phosphatase- and protease-inhibitor cocktails as described previously [40]. Cell lysates (30 μg total protein) were separated on 12% SDS–polyacrylamide gels and transferred onto a polyvinylidene fluoride membrane. After blocking with 5% non-fat milk for 1 h at room temperature, blots were incubated overnight at 4 °C with primary antibodies, followed by incubation with horseradish peroxidase-conjugated secondary antibodies for 2 h at room temperature. Protein bands were detected using an enhanced chemiluminescence detection kit (Pierce, Rockford, IL, USA).

### 4.8. APE1/Ref-1 Luciferase Reporter Assay

Primary VSMCs were co-transfected with an APE1/Ref-1-promoter–luciferase reporter construct (pGL3; −4000/+65 ,kindly provided by Kishir Bhakat, University of Nebraska Medical Center, Omaha, ME, USA), and a *Renilla* luciferase control (pRL-TK, Promega, Madison, WI, USA) at a 20:1 ratio using Effectene transfection reagent (Qiagen, Valencia, CA, USA) according to manufacturer instructions. The following day, the medium was changed to calcification medium, and transfected cells were cultured for an additional 4 days. Cells were washed with cold PBS and lysed with passive lysis buffer (Promega, Madison, WI, USA). Luciferase activity was measured using a dual luciferase reporter assay system (Promega, Madison, WI, USA) according to manufacturer instructions. Firefly luciferase activities were normalized to those of the co-transfected *Renilla* luciferase. 

### 4.9. Intracellular and Mitochondrial ROS

Intracellular ROS levels were measured using the fluorescent probe H_2_DCFDA (Invitrogen, San Diego, CA, USA). Cells were washed with HBSS and incubated with 5 μM H_2_DCFDA in HBSS at 37 °C for 30 min. The fluorescence intensity was measured at wavelengths of 485 nm for excitation and 530 nm for emission using a fluorescence microplate reader (Thermo Fisher Scientific, Waltham, MA, USA). Mitochondrial ROS levels were determined using the MitoSOX red mitochondrial superoxide indicator (Invitrogen, San Diego, CA, USA). Cells were washed with HBSS and incubated with 5 μM MitoSOX red in HBSS at 37 °C for 10 min, and fluorescence intensity was measured at wavelengths of 530 nm for excitation and 590 nm for emission using a fluorescence microplate reader (Thermo Fisher Scientific, Waltham, MA, USA).

### 4.10. LDH Activity 

To investigate whether VSMC calcification was apoptosis related, cytotoxicity was determined by measuring LDH activity in the cell-culture medium. After the indicated treatments, medium was collected, and LDH concentrations in the extracellular medium were examined using an LDH cytotoxicity detection kit (TaKaRa, Shiga, Japan) according to manufacturer instructions. Cell lysates were scraped, sonicated, and centrifuged at 15,000× *g* at 4 °C for 30 min. Protein concentration was measured using the Bradford assay (Bio-Rad, Munich, Germany), and cytotoxicity was normalized to protein concentration.

### 4.11. Statistical Analysis

Values are expressed as the mean ± standard error of the mean. Statistical evaluation was conducted using a one-way analysis of variance followed by Tukey’s post-hoc test. A *p* < 0.05 was considered statistically significant.

## Abbreviations

APE1/Ref-1	Aprinic/apyrimidinic endonuclease 1/redox factor-1
Pi	Inorganic phosphate
VSMC	Vascular smooth muscle cell
LDH	Lactate dehydrogenase
ALP	Alkaline phosphatase
Runx2	Runt-related transcription factor 2
Pit-1	Pituitary-specific positive transcription factor 1
α-SMA	α-Smooth muscle actin
SM22α	Smooth muscle protein 22-α
ROS	Reactive oxygen species
NF-κB	Nuclear factor-kappa B
FBS	Fetal bovine serum
MOI	Multiplicity of infection
DMEM	Dulbecco’s modified Eagle medium
H_2_DCFDA	2′,7′-dichlorofluorescin diacetate
siRNA	Small-interfering RNA
